# Medication‐induced obstructive uropathy and hyperprolactinemia in a pediatric patient

**DOI:** 10.1002/ccr3.2396

**Published:** 2019-08-29

**Authors:** Kwabena Nkansah‐Amankra, Sathyanarayan Sudhanthar

**Affiliations:** ^1^ Department of Pediatrics and Human Development College of Human Medicine Michigan State University East Lansing MI USA

**Keywords:** acute medicine, nephrology, pediatrics and adolescent medicine, pharmacology, psychiatry

## Abstract

Hyperprolactinemia can result from a pituitary tumor or decreased dopamine levels due to compression of the pituitary stalk. Hypothyroidism, renal failure, and drug interaction need to be ruled out as a part of diagnostic evaluation. The prolactin level often indicates the etiology, but drug interaction needs to be ruled out mainly in a patient who is on multiple medications.

**Learning points**

- Literature suggests hyperprolactinemia >200 ng/mL should raise suspicion about a possible tumor, but it is imperative to rule out drug interactions that may contribute to hyperprolactinemia.
- Checking drug interactions should be mandatory in a patient on multiple medications, especially psychotropic drugs, to rule out high prolactin levels.
- Exposure to radiation and cost associated with imaging can be saved if drug interaction is considered as a primary cause.

## 1 BACKGROUND

Prolactin, a hormone secreted by the anterior pituitary gland, has wide‐ranging effects on the body.^1^, ^2^, ^3^ Its primary role is in regulating the production of milk in females but has secondary functions, including pancreatic development and immune system and metabolism regulation.^1^, ^2^, ^4^ It is secreted in a pulsatile manner in reaction to activities including eating, mating, ovulation, estrogen treatment, and nursing.^1^, ^3^ During pregnancy, high levels of estrogen and progesterone cause a significant increase in circulating levels of prolactin. After childbirth, the sucking of the nipple by the baby prompts an increase in prolactin levels.^1^, ^3^ Prolactin levels also vary diurnally as high levels are found during REM sleep and in the early morning.

Hyperprolactinemia is defined as levels of prolactin above 18 ng/mL for men and 29 ng/mL for women.^1^ The etiology of hyperprolactinemia can result from decreased dopamine levels due to compression of the pituitary stalk or a pituitary gland tumor that results in increased secretion of prolactin.^2^ Drugs that can induce hypersecretion of prolactin include dopamine receptor blockers (risperidone, haloperidol, and metoclopramide), dopamine synthesis inhibitors (alpha‐methyldopa), catecholamine depletors (reserpine), and others. High prolactin levels inhibit gonadotropin‐releasing hormones, decreasing follicle‐stimulating hormone (FSH), and luteinizing hormone (LH) levels.^5^, ^6^ As such, patients with hyperprolactinemia present with oligomenorrhea, amenorrhea, or infertility in women and sexual dysfunction, visual problems, and headaches in all hyperprolactinemic patients. Hyperprolactinemia can also result in osteoporosis.^2^, ^6^, ^7^

Prolactin secretion is regulated by dopamine (a prolactin inhibitory hormone), which binds to D2 receptors on lactotrophs, reducing prolactin secretion from the anterior pituitary gland.^8^, ^9^ Thus, substances and conditions that can accentuate or decrease this activity of dopamine will result in hypoprolactinemia and hyperprolactinemia, respectively.^9^, ^10^ This paper explores the latter, looking at situations that arise in hyperprolactinemia, specifically drug‐induced hyperprolactinemia, by presenting the case of a 17‐year‐old male on multiple psychotropic medications found to have high prolactin levels.

Very high prolactin levels (>200 ng/mL) are often attributed to pituitary tumors. However, very rarely, they are shown to be due to drug interactions. We present an interesting patient case that of a 17‐year‐old male on Haloperidol, Benzatropine, and Lithium with hyperprolactinemia and obstructive uropathy. The combination of these medications was due to his mental health condition and is managed by a psychiatrist. This patient was diagnosed with levels of prolactin that are rarely seen in drug‐induced hyperprolactinemia. As far as we know, the combination of Lithium, Benzatropine, and Haloperidol has been seldom shown to lead to both hyperprolactinemia and obstructive uropathy.

## 2 CASE PRESENTATION

The patient presented in this report is a 17‐year‐old male with a past medical history of mild cerebral palsy, autism spectrum disorder, and bipolar disorder with aggression, which was being treated with Lithium and Haloperidol for his mental health symptoms over the past 9 months. Over the course of his treatment, he subsequently developed hypothyroidism due to Lithium and was started on Levothyroxine. He was started on Benzatropine as well for prophylaxis against dystonic movement and tremor issues known to be caused by Haloperidol.

During his annual well check, he was found to have a suprapubic mass due to the bladder distension and urinary retention. The patient has had a history of urinary retention on and off for a few months. Renal labs and ultrasound of the kidneys and bladder were ordered immediately along with a referral to urology. His initial labs showed that he had developed acute kidney injury (AKI) with high blood urea nitrogen (24 mg/dL) and increasing creatinine (2.4 mg/dL). The AKI was due to the chronic intermittent urinary retention patient had been experiencing for at least the past 4 months. At the urology visit, the plan was made to relieve his obstructive uropathy with intermittent catheterization and to coordinate with his psychiatrist and Primary Care Physician (PCP) to manage his mental health medications.

Two days after, the urologist saw him, he presented with a febrile illness to the emergency and was admitted to the pediatric Intensive care unit due to his labs, which showed a urinary tract infection, metabolic acidosis, hyponatremia, and toxic levels of Lithium. A prolactin level was also checked since he was on haloperidol and he was found to have galactorrhea on physical exam. His Lithium level (2.2 ng/mL) was in a toxic range, and his prolactin levels were very high, leading to suspicions of a pituitary tumor (267 ng/mL). He was also diagnosed with grade 3 bilateral hydroureteronephrosis with obstructive uropathy as per the ultrasound.

Due to the suspicion of a pituitary tumor, he underwent an MRI imaging of his brain after his creatinine normalized. The MRI showed no abnormality, ruling out a pituitary adenoma. During the subsequent hospital course, Benzatropine, Haloperidol, and Lithium were held until the patient's renal function was normalized. He was later discharged on lower Lithium, Benzatropine, and Haloperidol doses. His prolactin levels were still high at the time of his discharge from the hospital. Urology was consulted in the hospital, and an aggressive bladder program was initiated with close follow‐up planned with his primary care, urologist, and psychiatrist.

### 2.1 Differential diagnosis

The differential diagnoses of hyperprolactinemia clinically depend on the level of prolactin.

Low levels of hyperprolactinemia have found to be due to medications, hypothyroidism, and kidney problems. Clinical judgment, when encountered with higher levels of hyperprolactinemia (>200 ng/mL), calls for ruling out pituitary adenoma. Our patient had many medications that could potentially interact with each other. Due to his initial clinical presentation of cerebral palsy, autism spectrum disorder, and bipolar disorder, this patient was a challenging dilemma in terms of drug prescription. The most likely explanation of the events is that Lithium led to hypothyroidism and hypothyroidism leads to an increase in TRH, which stimulates prolactin release. Our patient was also in combination with Haloperidol and Benzatropine, and the combined anticholinergic effects led to chronic urinary retention, which culminated in AKI, leading to toxic levels of Lithium. Increased Lithium level, in combination with Haloperidol effect on dopamine, is what we suspect led to his hyperprolactinemia.

### 2.2 Outcome and follow‐up

**Figure 1:**
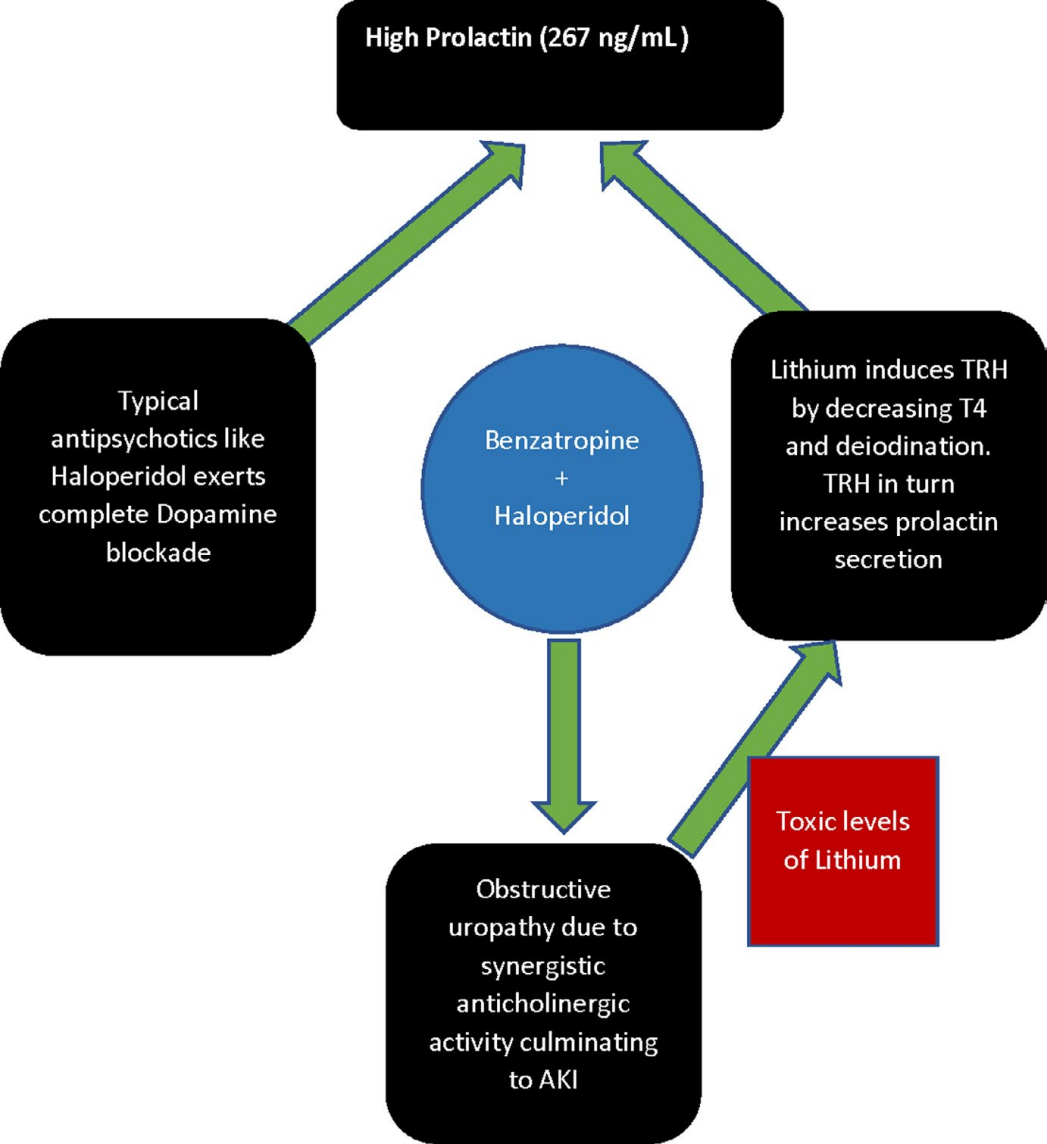
Demonstration of drug interaction which led to the high prolactin levels in our patient. Combination of Benzatropine and Haloperidol caused obstructive uropathy in this patient, which culminated in AKI, resulting in toxic levels of Lithium causing hyperprolactinemia along with the independent effect of haloperidol

The patient’s prolactin levels normalized over 8 weeks. He continued to be on optimal doses of all three medications and was managed carefully by the psychiatrist. He also underwent a procedure for intermittent suprapubic catheterization, which would address the chronic issue of urinary retention. This is widely believed to be due to the synergistic anticholinergic effects of Haloperidol and Benzatropine, which contributed to obstructive uropathy, causing AKI, which in turn increased Lithium levels in the body, culminating in increased levels of prolactin (Figure 1). Lithium is renally cleared, whereas Haloperidol goes through extensive first‐pass metabolism through the liver. One of the main side effects reported when using Lithium with neuroleptics is there is an increased chance of neurotoxicity in the form of confusion, tremor, extrapyramidal symptoms, neuroleptic malignant syndrome, and QT prolongation.^11^, ^12^, ^13^, ^14^ One of the possible mechanisms of increased neurotoxicity when using Lithium and the neuroleptic combination is that neuroleptics can increase the intracellular concentration of Lithium.^15^ Other mechanisms include enhanced dopamine blockade (Risperidone), increased intracellular Lithium levels (Risperidone), and interaction of serotonergic effects of Clozapine with Lithium. This case highlights the importance of monitoring drug dosages, especially when there are multiple drugs involved, as synergistic and antagonistic drug‐drug interactions can be detrimental to the health of the patient.^15^

## 3 DISCUSSION

Current literature shows that anticholinergic and alpha‐adrenergic drugs lead to urinary retention and dopamine antagonists cause hyperprolactinemia. However, the combination of an anticholinergic drug and dopamine antagonist resulting in urinary retention and subsequent hyperprolactinemia, as in this patient case, has not been reported.^1^, ^2^

High prolactin levels associated with antipsychotics are quite common. In a literature review, it was found that 60% of women and 40% of men treated with prolactin‐sparing antipsychotic had a prolactin level above the upper limit of the normal range.^16^ In a study on the prevalence of hyperprolactinemia in schizophrenic patients, it was found that the mean serum level was 41.5 ng/mL with 69% of the patients having levels above normal.^17^ Risperidone was found to be especially guilty of increasing prolactin levels, with the prevalence of hyperprolactinemia was 88% among females taking risperidone versus 47.6% of those taking conventional antipsychotic drugs.^18^ A study that measured prolactin levels and clinically significant sexual dysfunction found that in switching from risperidone to paliperidone palmitate, a fourfold reduction in the measurable parameters was seen.^19^

Further studies support this claim, as young males treated with risperidone reported diminished sexual function in comparison to those not treated with antipsychotics.^20^ Haloperidol shows similar effects to risperidone, as one study reported a high of 77 ng/mL in a study population.^21^ Very high prolactin levels (>200 ng/mL) are often attributed to tumors.^2^ However, rarely, they are shown to be due to antipsychotics. This is what makes the current case so interesting: that of a 17‐year‐old male on Haloperidol reporting with levels of prolactin that are rarely seen in drug‐induced hyperprolactinemia.

This case highlights the importance of understanding how drug‐drug interactions can upset the physiological balance in a patient. The patient was on drugs that are rarely used in combination, and their subsequent synergistic effects contributed to his presentation. This drug combination, combined with his unique presentation of obstructive uropathy, leading to hyperprolactinemia offers an insight into understanding dosage prescription and drug‐drug interaction. It is multidisciplinary, tying in multiple disciplines such as physiology, pharmacology, endocrinology, and nephrology.

## CONFLICT OF INTEREST

None declared.

## AUTHOR CONTRIBUTIONS

All the authors were involved in patient care when the patient was scheduled for regular follow‐ups. KN wrote the first draft of the manuscript, and SS was involved in the revision of the manuscript and final submission. SS was also involved in getting consent from the family and patient. SS and KN prepared a poster presentation of this case report for the regional medical conferences.
